# The impact of patient- and family-centered care interventions on intensive care unit outcomes: a meta-analysis of randomized controlled trials

**DOI:** 10.1016/j.bjane.2024.844577

**Published:** 2024-11-26

**Authors:** Yangjin LV, Peng Li, Ronghui Li, Ting Zhang, Kaifang Cai

**Affiliations:** a Department of Intensive Care Unit, Hangzhou Red Cross Hospital, Hangzhou, Zhejiang, China; b Yiyang Medical College Nursing Department, Yiyang, Hunan, China; c Operating Room of Linyi Tumor Hospital Linyi City, Shandong Province, China; d Ankang Central Hospital, Department of General Surgery, Ankang Shaanxi, China; e Ankang Central Hospital, Department of Nursing, Ankang Shaanxi, China

**Keywords:** Critical care, Delirium, Intensive Care Unit, Meta analysis, Patient centered care

## Abstract

**Background:**

Patient and Family-Centered Care (PFCC) interventions are increasingly recognized as a viable approach to address various mental health issues among patients in Intensive Care Units (ICUs). Therefore, this review aims to estimate the effect of Patient and Family-Centered Care Interventions on specific outcomes in adult patients admitted to Intensive Care Units (ICUs).

**Methods:**

We systematically searched four major databases for parallel arm Randomized Controlled Trials (RCTs). The PRISMA framework was used to report our review. We included studies involving adult patients (> 18-years) admitted to ICUs and examined the effects of any type of Patient and Family-Centered Care intervention (PFCC) on outcomes such as depression, anxiety, delirium, and length of hospital stay. Data extraction was performed independently by two authors in Medline, Google Scholar, and ScienceDirect, from inception to July 2024. Random effects model was used to pool the data.

**Results:**

A total of 11 studies were included in our systematic review and meta-analysis, with a combined sample size of 3352 patients (PFCC group, n = 1681; usual care group, n = 1671). A random-effects model revealed a significant reduction in delirium prevalence in the PFCC group, with a pooled Risk Ratio (RR) of 0.54 (95% CI 0.36 to 0.81). However, no statistical significance was found for other outcomes such as depression, length of ICU stay, and anxiety. It is important to note that all the included studies were assessed to have either a high or unclear risk of bias.

**Conclusion:**

PFCC interventions may significantly reduce delirium rates among ICU patients; however, their effects on other outcomes, such as depression, anxiety, and length of stay, were not statistically significant.

## Introduction

Admission to an Intensive Care Unit (ICU) due to critical or terminal illness is a stressful experience for both patients and their relatives.^1^^,^^2^ These stressful situations have been shown to increase physical and psychological stress among patients, leading to delirium, depression, and anxiety.^3^ Studies have even demonstrated that these events further complicate ICU admissions, resulting in longer stays, increased post-traumatic stress, and even mortality.^3^ Recent research has also highlighted the impact of these consequences on the families and relatives of patients, who may experience anxiety, depression, and Post-Traumatic Stress Disorder (PTSD) during their time in the ICU.^4^^,^^5^

The Institute of Patient and Family-Centered Care defines family-centered care as an approach that involves planning, evaluation, and the delivery of mutually beneficial care between healthcare providers, patients, and their family members.^6^ PFCC interventions are based on four core principles: dignity and respect, information sharing, patient and family participation in care, and collaboration between healthcare providers and families.^7^ This approach emphasizes the involvement of family members in the delivery of healthcare.^8^ Recent studies have suggested that PFCC can be a viable intervention for improving psychological and clinical outcomes, given they empower patients, improve coping mechanisms and satisfaction, prominently reduce psychological distress and streamline the care process.^9^, ^10^, ^11^ As a result, PFCC has become an integral part of hospital care, encompassing the implementation of management protocols, counseling, and specific interventions.^12^ In an ICU setting, effective patient and family communication is a crucial challenge, as we heavily rely on families for decision-making and support.^13^ However, some studies have raised concerns about the potential for PFCC to contribute to additional cases of delirium among patients and family members. As of now, there has been no comprehensive review examining the effects of PFCC on ICU outcomes, specifically delirium, depression, anxiety, and mortality. Therefore, we conducted this review to estimate the impact of Patient and Family-Centered Care Interventions on specific outcomes in adult patients admitted to intensive care units.

## Methods

### Research question

Among adult patients admitted to the Intensive Care Unit, what is the effect of Patient and Family-Centered Care Interventions on ICU outcomes?

### Inclusion and exclusion criteria

For this review, we included all parallel-arm individual randomized, or cluster randomized controlled trials. Studies reported as full text were included, while studies published only as abstracts or unpublished data were excluded. We followed the Preferred Reporting Items for Systematic Reviews and Meta-Analyses (PRISMA) framework to report our results.^14^ We only included studies reported in English from the following databases: Medline, Google Scholar, and ScienceDirect, from inception to July 2024. The detailed search strategy is explained in the supplemental material.

### Participants

We included studies with adult patients (> 18-years) admitted to ICUs, assessing the impact of any PFCC interventions on outcomes like depression, anxiety, delirium, and length of hospital stay. We excluded studies conducted among pediatric and neonatal patients and specialized ICUs. A family member, defined based on individual studies, could be an adult (> 18-years), spouse, partner, friend, or blood or non-blood relative of the adult ICU-admitted patients.^15^

### Type of intervention and comparator

Our review included studies involving any type of Patient and Family-Centered Care Interventions (PFCC) aimed at improving outcomes in adult ICU patients. We included studies that provided PFCC to adult patients regardless of their disease severity. We excluded studies that focused on interventions for family members, interventions implemented before improvement in study outcomes prior to ICU admission, interventions targeting specific subgroups such as dying patients or patients with cognitive impairment, and interventions that did not involve the active participation of family members during their ICU stay. The intervention was compared to a control group receiving standard medical care without any form of PFCC intervention during their ICU stay.

### Type of outcome measure

The primary outcome of interest was to examine the difference in the rate of depression in the intervention and comparator group. Secondary outcome were anxiety, delirium and length of hospital stay (in days).

We included studies reporting any of the above-mentioned outcomes in either arm.

### Data collection and analysis

Two independent investigators (YL and PL) conducted the literature search, and screened titles, abstracts, and keywords of all selected studies for inclusion in our review. Relevant full-text articles were extracted. The abstracts and full texts of retrieved articles were further screened independently by the primary and secondary investigators. We used the PICOS (Population, Intervention, Control, and Outcome Study) design for identifying and extracting potential studies for inclusion in our analysis. Any disagreements between the two investigators during the selection process were resolved through consensus or consultation with a third investigator (KC). The third investigator also monitored the quality of the overall review process.

The primary investigator extracted relevant study characteristics for the review from the included studies. Only the relevant arms were included for studies reporting multiple arms from a single trial. The primary investigator entered the data into Microsoft Excel, and the third investigator double-checked the data entry for accuracy.

Two independent investigators (RL and TZ) assessed the risk of bias in the included studies using the Cochrane risk of bias tool for Randomized Controlled Trials.^16^ Randomization was used for the allocation of study participants into intervention and control arms in all the included studies. However, none of the included studies provided a clear description of blinding of participants and outcome assessment. Five out of the eleven included studies had a high risk of bias with respect to blinding and were categorized as high risk, while the remaining six were categorized as an unclear risk due to insufficient information on blinding and outcome assessment. We summarized the risk of bias using the criteria established by Higgins et al.^17^

### Statistical analysis

We evaluated the pooled effect of PFCC on the outcome such as a change in the rate of delirium, anxiety, depression, and length of ICU stay through the inverse variance method using the Mean Difference (MD) and standard deviation. Binary outcomes such as delirium were summarized using Risk Ratios (RR) with a 95% Confidence Interval (95% CI) using the Mantel-Haenszel method. Finally, the pooled estimate was reported as mean difference with 95% Confidence Interval. Analysis was performed in STATA version 14.2.

### Assessment of heterogeneity

Chi-Square test of heterogeneity and I^2^ statistic (to quantify heterogeneity) were used to assess the between-study variance due to heterogeneity. I^2^ less than 25% was considered mild, 25%–75%, moderate, and more than 75% as substantial heterogeneity. Study details and pooled estimates were graphically represented through a forest plot. Heterogeneity often indicates significant variability in study design, populations, interventions, or other factors.

Since we collected information from openly available sources, we do not warrant an Ethical clearance

### Certainty of evidence

The quality of evidence in the included studies was evaluated following the Grading of Recommendations Assessment, Development, and Evaluation (GRADE) framework. As per GRADE guidelines, Randomized Controlled Trials (RCTs) initially provide high-certainty evidence. However, the certainty of evidence was downgraded based on our evaluation of five key domains: study limitations, inconsistency, imprecision, indirectness, and publication bias. For each outcome, the evidence was classified as high, moderate, low, or very low certainty. Additionally, the GRADE criteria account for both the certainty of evidence and the magnitude of the observed effect.

## Results

### Study selection

**Figure 1:**
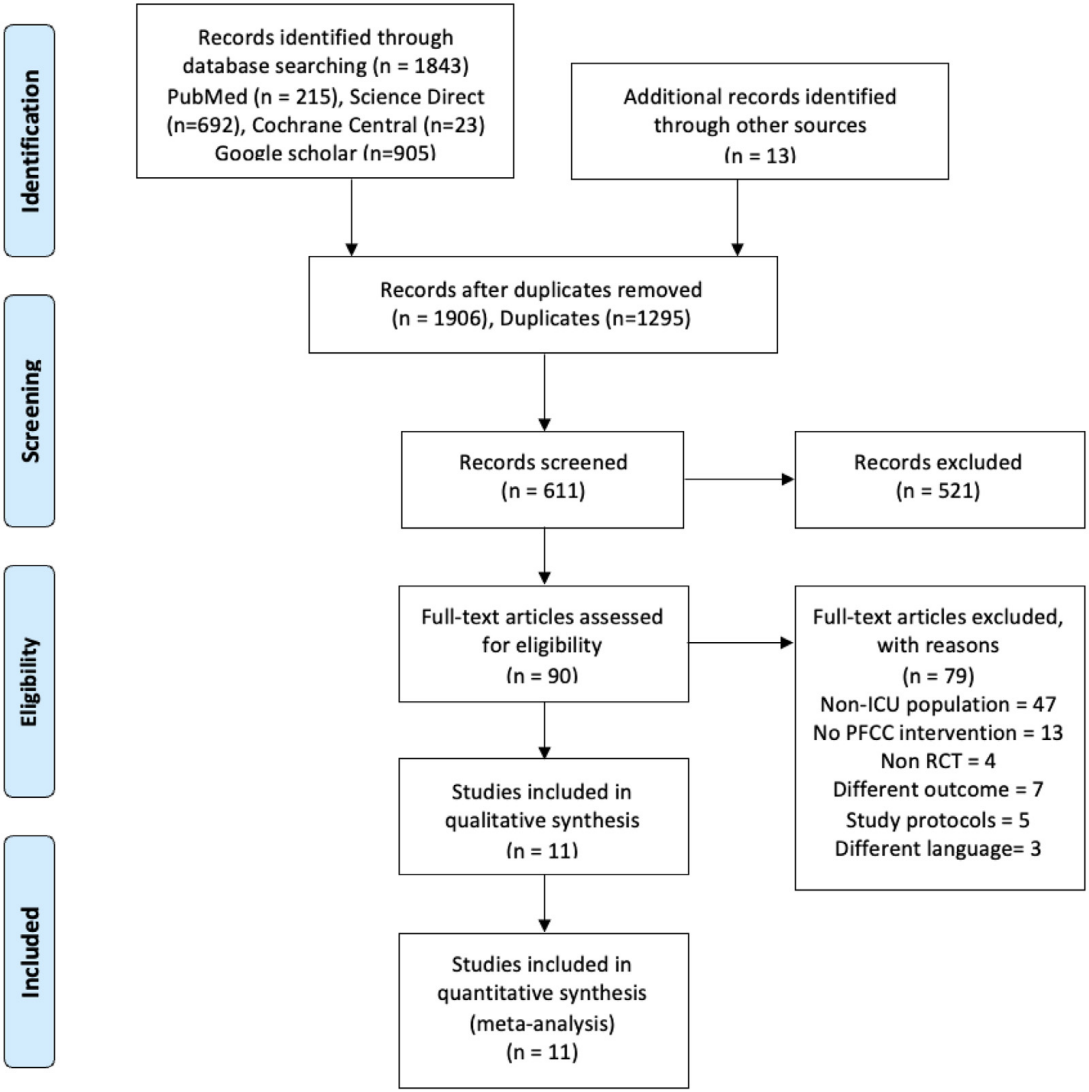
PRISMA flow diagram explaining the search flow.

Through our systematic review, a total of 1906 articles were identified and screened, of which 1295 duplicates were removed. During the primary screening, 521 articles were excluded as they did not meet our inclusion criteria. Thus, finally, 90 articles were chosen for secondary screening, of which 11 were included for the systematic review and meta-analysis.^18^, ^19^, ^20^, ^21^, ^22^, ^23^, ^24^, ^25^, ^26^, ^27^, ^28^ The total number of participants was n = 3352, with the PFCC group consisting of n = 1681 and the usual care group consisting of n = 1671. The PRISMA flow diagram explaining the process is presented in Figure 1.

### Included and excluded studies

Table 1 provides the study characteristics of the individual articles included in the review. The intervention mainly focused on any intervention that included Patient and Family-Centered Care Interventions (PFCC) aimed at improving outcomes of adult patients in the ICU. The most commonly studied primary outcome was depression, anxiety, and delirium, while other commonly studied secondary outcomes included in-hospital mortality and length of ICU stay. Sample sizes across these studies ranged from as small as 35 participants (Sayde 2020 in the USA) to as large as 1685 participants (Rosa 2019 in Brazil). The interventions typically involved diaries, educational booklets, and structured communication between healthcare providers, patients, and their families, aiming to reduce psychological stressors such as anxiety, depression, and delirium during and after ICU stays. The primary outcomes measured across the studies largely focused on psychological effects, particularly the reduction of delirium, anxiety, and depression, with tools like the Hospital Anxiety and Depression Scale (HADS) and Confusion Assessment Method for ICU (CAM-ICU) commonly employed. Secondary outcomes frequently included length of ICU stay and overall hospital stay, although reductions in these outcomes were not uniformly observed across all studies.

**Table 1:** Characteristics of included studies (n = 11).

Study and study design	Country	Study population	Sample size	Type of ICU and number of sites	Primary and Secondary outcomes	Description
Garrouste 2019 (RCT)	France	Patients aged more than 18-years on mechanical ventilation for > 48 hours, who were initiated on ventilation within 48 hours of ICU admission	n = 339 (Intervention = 164, Control = 175)	Multicentric study including 35 ICUs	Primary outcomes: The proportion of patients who had PTSD symptoms after 3-months of ICU discharge, evaluated through Impact of Event Scale-Revised (IES-R) questionnaire. Secondary outcomes: Secondary outcomes measured included anxiety and depression measured using HADS (Hospital Anxiety and Depression Scale (HADS) score) evaluated 3-months after ICU discharge and length of ICU stay	The intervention was basically a diary that was filled out duly by the clinicians and the family members, while the concluding note was written by the clinician before patients were transferred to the ICU. The diary details were explained to the patients before they were discharged and in case, they were not able to comprehend it or understand it, it was detailed to the family member or the caretaker. The comparator group did not receive any diary intervention.
Bench 2015 (Cluster RCT)	United Kingdom	Patients over 18-years of age who were at least 72h in the ICU and declared fit to be transferred to the general ward.	n = 158 (Intervention - user-centered critical care discharge information pack (UCCDIP; n = 51), control 1: ad hoc verbal information (n = 59), control 2: booklet published by ICU steps (n = 48).	A multicentric study including 2 ICUs with mixed patients (medical, surgical and trauma care)	Primary outcomes: Patient Anxiety and depression were measured using HADS (Hospital Anxiety and Depression Scale-HADS score) post 28-days of ICU stay. Secondary outcomes: Length of Stay (LOS).	The patients were provided with discharge information booklets which either had UCCDIP or the ICU steps; one each for the patient and the family prior to discharge. The booklet had information regarding discharge summary, diary pages to write about patient's thoughts and feelings that they felt during the hospital stay. The comparator group received the usual care with adhoc verbal information on ICU discharge
Sayde 2020 (RCT)	USA	The study included patients who were over 18-years of age and had an ICU stay of more than 72h, and also who were sedated and intubated for more than 24h, with any pre-existing PTSD, dementia, or intracranial injury	n = 35 (Intervention = 18, Control = 17)	Single, mixed ICU with both medical and surgical patients	Primary outcomes: Post traumatic stress measured using IES-R, general well-being using the Patient Health Questionnaire (PHQ-8) Anxiety and depression using Hospital Anxiety and Depression Scale (HADS), and Generalized Anxiety Disorder 7-item (GAD-7) at 1-week of ICU discharge, and again at 4-weeks, 12-weeks and 24-weeks following discharge. Secondary outcomes: Length of hospital stay	All patients who were enrolled into the intervention group were provided with a bedside diary, and the patients and family were asked to write down the changes and daily events. PTSD education was provided to the participants starting within 1-week of admission.
Azoulay 2018 (RCT)	France	Patients were enrolled into the study if they were > 18-years and had received mechanical ventilation before 48hrs of ICU admission. For every patient one relative was included.	n = 302 (Intervention = 148, Control = 154)	Multicentric study with included data from 14 ICUs	Primary outcomes: Anxiety and depression measured using HADS after 5-days of intervention. Secondary outcomes: Family satisfaction	Validated list of 21 questions were given to relatives so they could ask questions to help improve comprehension of patient diagnosis and treatment. Usual care had family and staff conferences with no intervention list.
Black 2010 (RCT)	Ireland	Patients were enrolled into the study if they are > 18-years and provided consent for the study Patients and family members who were not able to participate physically in the intervention were excluded.	n = 170 (83 in the control group and 87 in the intervention group)	A single center study including mixed ICUs	Primary: Intensive Care Delirium Screening Checklist (ICDSC) was used to measure delirium, Therapeutic Intervention Scoring System-28 to measure severity of illness, and Sickness Impact Profile for psychological recovery measured on day 1, 14 and at months 1, 2 and 3.	The intervention was provided to all patients in the intervention group through voice messages that were aimed to reduce the stressors during the stay in ICU. The intervention included a booklet that contained information on delirium guide and psychological care. Information on how to deliver the information verbally and written ways were outlined and explained to the intervention group. Control group received usual care.
Gan Xioaqing 2017 (RCT)	China	Patients were enrolled into the study if they were > 18-years and who provided consent for the study with no previous history of diagnosed delirium	n = 391 (178 in the control group and 213 in the intervention group)	A single center study including mixed ICUs	Primary: Delirium measured during follow up after discharge and 14-days after, measured using Confusion Assessment Method–ICU (CAM–ICU) tool. Secondary outcomes: Length of hospital stay	The intervention group received a booklet that had instructions to the patient and the family member on prevention of delirium. The control group received usual care.
Jiao Xueping 2021 (RCT)	China	Patients were enrolled into the study if they were > 18-years and who provided consent for the study with no previous history of diagnosed delirium	n = 164 (82 in the control group and 82 in the intervention group)	A single center study including mixed ICUs	Primary: Delirium measured during follow up after discharge and 14-days after, and post 3-months of discharge. Secondary outcomes: Length of hospital stay	The intervention group received a diary that was provided to note down the daily happenings and proceedings by the patient, and by the family member, in case the patient. Health education on delirium and anxiety prevention was provided to them three days post admission in ICU. The control group received usual care.
Eghbali Babadi 2017 (RCT)	Iran	Patients between 18‒70-years, who were accompanied by their family members, with no history of addictions, delirium, cognitive or mental disorders	n = 68 (34 in both control group and intervention group)	A single center study including patients who were admitted following open heart surgery in the ICU	Primary: Development of delirium, assessed using the Confusion Assessment Method–ICU (CAM–ICU) tool designed to assess the patients concerning delirium (twice a day assessed). Secondary outcomes: Level of irritability measured using the Richmond Agitation Sedation Scale (RASS)	The next day after surgery, one among the family members in the intervention group who received education (which focused on the goal, signs, causes and methods of prevention of delirium) prior visited the patient. Pamphlets were also distributed to them. In the control group, patients received routine care and no special visit was made
Ma Hong 2015 (RCT)	China	Patients above 18-years of age, who were admitted for more than 72 hours in ICU, with no prior diagnosed delirium or cognitive dysfunction	n = 164 (Intervention = 84 and Control = 80)	A single center study including patients from mixed ICU	Primary outcomes: Delirium rates measured using CAM-ICU	The intervention included a booklet that was provided to the family member, that consisted of educational information on guidance to alleviate stress response and improve psychological stability. In the control group, patients received routine care
Mitchell 2017 (RCT)	Australia	The study participants included patients aged more than 16-years, with more than 96 hours ICU stay, and willing to get screened for delirium, and a family member attending them. One family member per patient was selected	434	A single center study including patients from mixed ICU	Primary: Delirium rates measured using CAM-ICU. Secondary outcomes: Level of irritability measured using the Richmond Agitation Sedation Scale (RASS)	The intervention consisted of an educational protocol that had the template on description of intervention and a guide that helps in implementation which was adopted from previous studies. The protocol had details on orientation on memory, therapeutic engagement for cognitive stimulation and has activities to discuss family events, finally a sensory tool which has hearing aids and glasses. The control group received only usual care.
Rosa 2019 (Cluster randomized trial)	Brazil	Patients more than 18-years admitted to the ICUs with more than 96 hours stay were included.	n = 1685 (Intervention = 837 and Control = 848)	Cluster-crossover RCT including 36 mixed ICUs	Primary: Delirium rates measured using CAM-ICU. Secondary outcomes: Length of ICU stay, ICU acquired infections	The intervention included a model through which the flexible visits to ICU were advocated for family education. Family members attended the meeting regularly to learn about ICU environment, palliative care and delirium prevention.

Out of the 76 full-text articles that we extracted; we excluded 65 studies. Among them, 42 were conducted among non-ICU populations, 13 had no clear definition of PFCC, four were non-Randomized Controlled Trials (non-RCTs), three had different outcome measures, two were study protocols, and the remaining one was in Arabic.

### Effects of interventions

**Figure 2:**
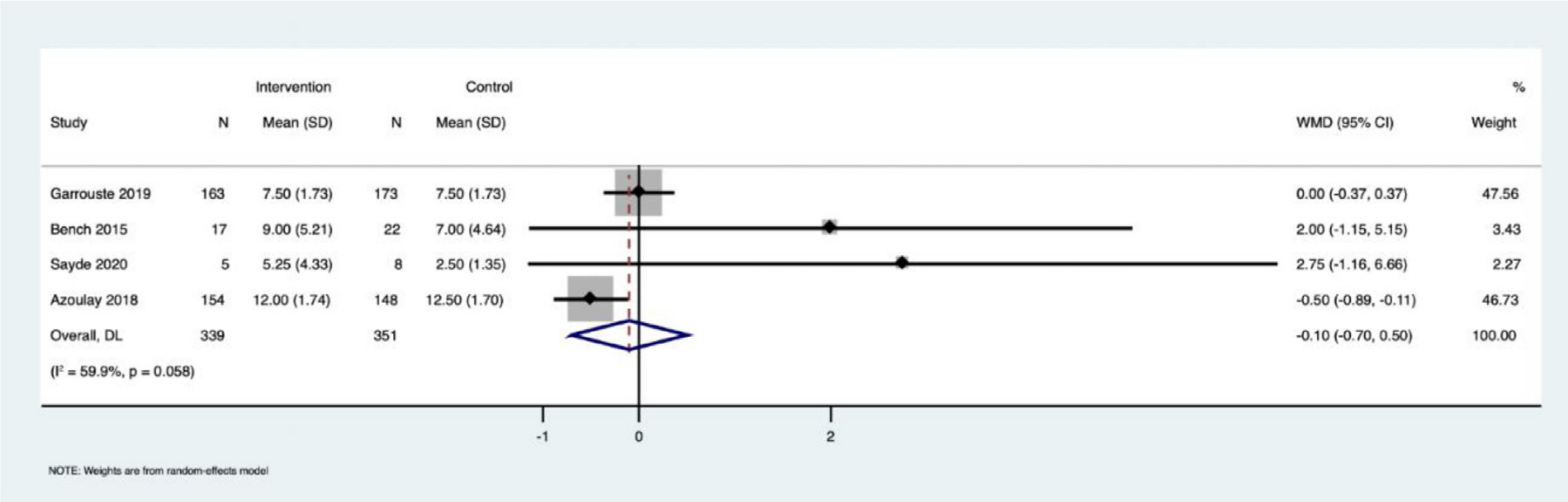
Forrest plot describing the effect of PFCC on anxiety among patients admitted in ICU.

Effect of PFCC on anxiety among patients admitted to the ICU: Four studies reported anxiety among patients (n = 690, PFCC group: n = 339, control group: n = 351). The Pooled Mean Difference (PMD) was estimated as -0.10 (95% CI: -0.70 to 0.50), indicating no significant reduction in anxiety levels across the groups (Fig. 2). We found moderate heterogeneity among the studies in reporting this outcome (I^2^ = 59.9%, p = 0.058). The data was extracted as mean (SD) from all four studies that utilized the Hospital Anxiety and Depression Scale (HADS) questionnaire to measure anxiety. Another study by Neilson et al. was not included in the analysis as it failed to report the mean (SD) of the depression and anxiety scores between the groups, reporting anxiety as a binary outcome with a cut-off > 11 as anxiety.^29^

**Figure 3:**
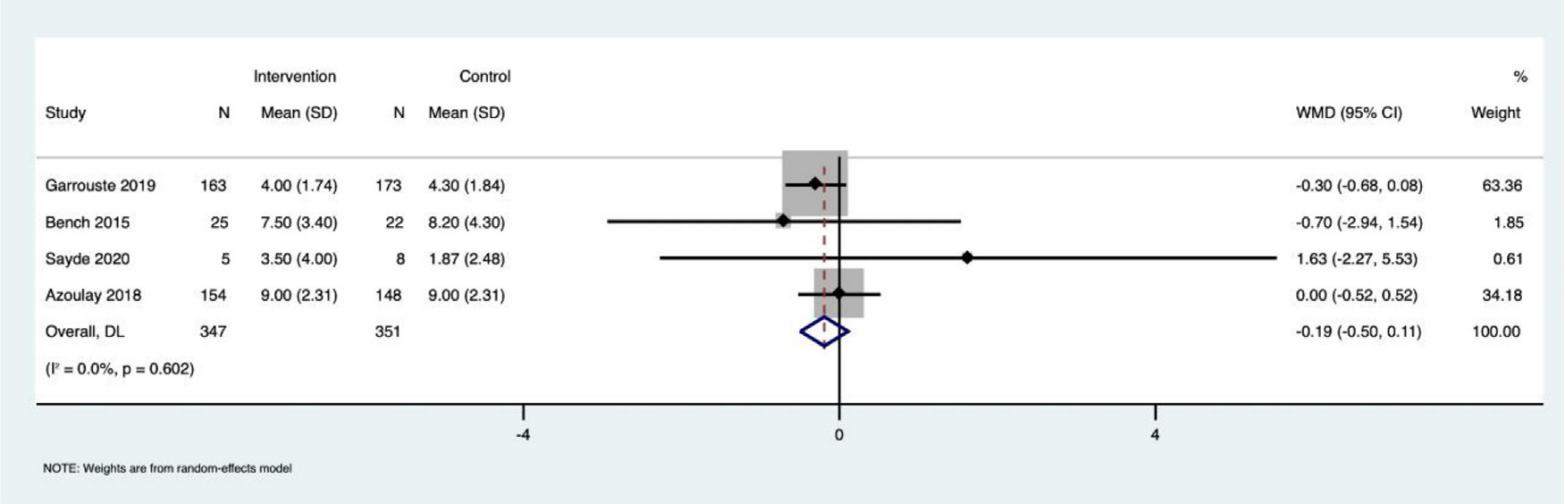
Forrest plot describing the effect of PFCC on depression among patients admitted in ICU.

Effect of PFCC on depression among patients admitted to the ICU: The same four studies that reported anxiety among patients (n = 690, PFCC group: n = 339, control group: n = 351) also reported depression measured using the same HADS questionnaire. The pooled mean difference (PMD) was -0.19 (95% CI: -0.50 to 0.11), indicating that PFCC did not significantly reduce depression between the intervention group and the control group (Fig. 3). We found nil heterogeneity among the studies in reporting this outcome (I^2^ = 0.0%, p = 0.60).

**Figure 4:**
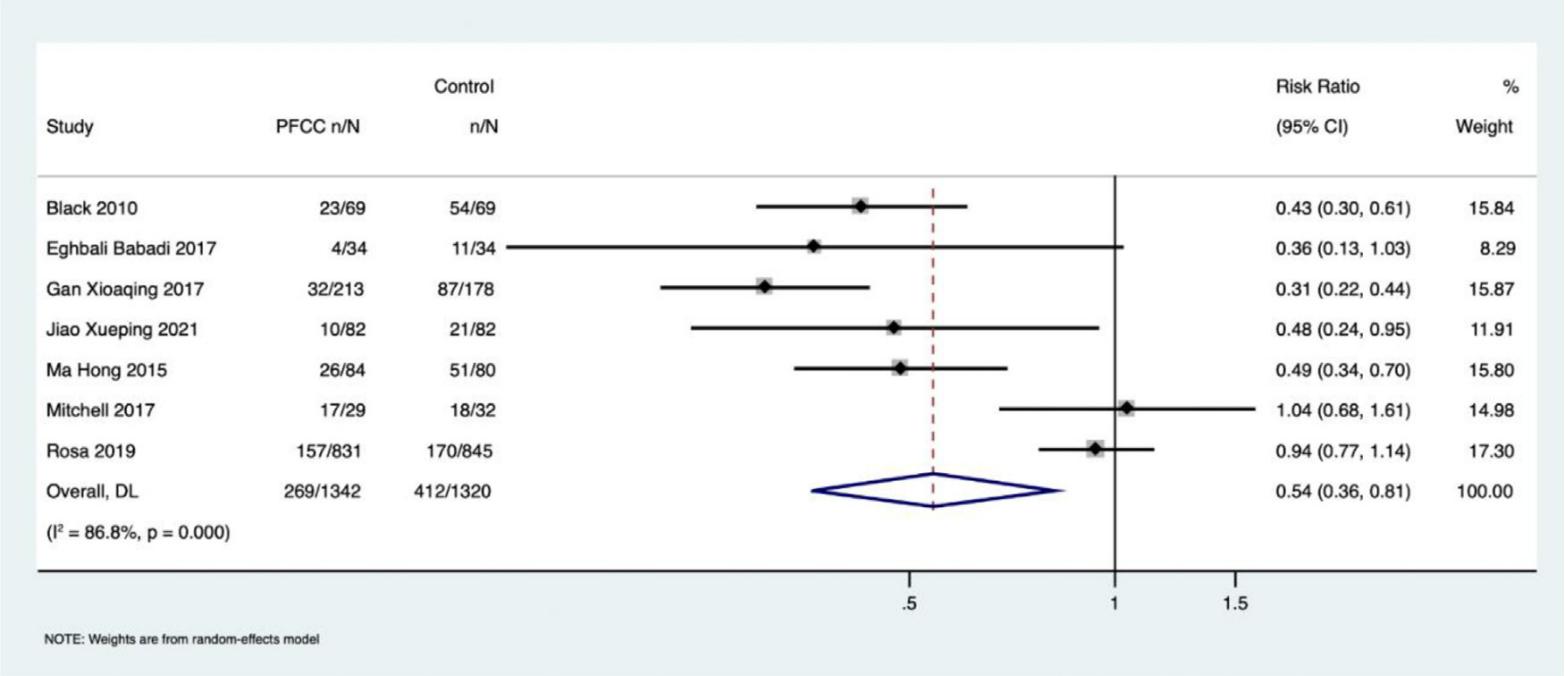
Forrest plot describing the effect of PFCC on delirium among patients admitted in ICU.

Effect of PFCC on delirium among patients admitted to ICU: Of the 11 included studies, seven reported delirium prevalence as an outcome measure at the study endpoint (n = 2662; PFCC group: n = 1342; control group: n = 1320). Using the random-effects model, we observed a significant reduction in delirium prevalence in the PFCC group, with a pooled Risk Ratio (RR) of 0.54 (95% CI 0.36 to 0.81), although there was significant heterogeneity among the studies (I^2^ = 86.8%, p < 0.001) (Fig. 4).

**Figure 5:**
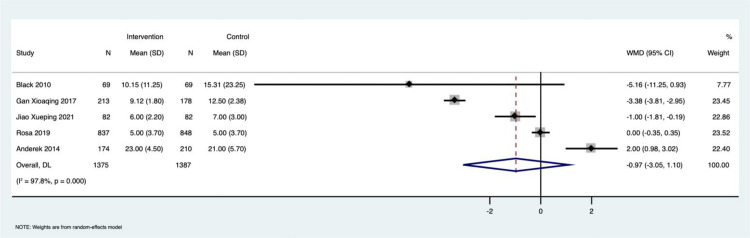
Forrest plot describing the effect of PFCC on length of ICU stay among patients admitted in ICU.

Effect of PFCC on length of ICU stay among patients admitted to the ICU: Five studies reported the change in the duration of ICU stay for both groups (n = 2762; PFCC group: n = 1375; control group: n = 1387). The Pooled Mean Difference (PMD) was -0.97 (95% CI: -3.05 to 1.10), indicating no significant reduction in ICU stay duration among the groups (Fig. 5). We found very high heterogeneity among the studies in reporting this outcome (I^2^ = 97.8%, p < 0.001).

### Sensitivity analysis

We did not perform a sensitivity analysis as we did not find any low-risk studies included in our review. Out of the eleven studies included in our review, five had a high risk of bias, while the remaining six had an unclear risk of bias. GRADE evidence showed that all studies included in our review were of low quality of evidence.

## Discussion

In our systematic review and meta-analysis, we pooled findings from various studies conducted across countries to estimate the effect of Patient and Family-Centered Care Interventions (PFCC) on specific outcomes among adult patients admitted to Intensive Care Units (ICUs). We included eleven Randomized Controlled Trials (RCTs) to evaluate the impact of PFCC on outcomes such as patient depression, anxiety, delirium, and length of hospital stay. However, we only observed a significant reduction in ICU stay in the PFCC group compared to the control group.

The ICU environment is known to induce fear and emotional distress among family members of admitted patients due to factors like inaccessibility, lack of information, absence of joint decision-making, and uncertainty of life.^30^ Frequent communication and involving family members in the healthcare decision-making process can enhance family understanding of the patient's health condition and provide hope. Additionally, including family members in the decision-making process can improve risk communication by healthcare personnel.^31^

Previous studies have shown that ICU-related delirium primarily stems from fear of the ICU environment and patients’ medical condition. Furthermore, patients without family visits tend to have a threefold higher risk of developing delirium.^32^ While previous studies have demonstrated the effectiveness of PFCC interventions in preventing delirium among non-ICU populations, they did not observe a significant change in the duration of delirium. Our study, on the other hand, showed that PFCC had a significant impact on reducing delirium in ICU patients. Another interprofessional multinational perspective document by Pandharipande in 2017 advocates for RCTs exploring the effect of PFCC on delirium reduction.^33^ Our results align with previous studies by Lin et al. and McKenzie et al.^34^, ^35^, ^36^

In terms of depression and anxiety reduction, our study did not observe statistically significant results through PFCC intervention. However, contrasting results were shown in studies by Neilson et al. and Sayde et al.^18^^,^^29^ These discrepancies could be attributed to differences in study population, comorbidity patterns, intervention types and durations, as well as variations in outcome assessment scales. Our findings are consistent with a meta-analysis conducted by Bohart et al. in 2021, which similarly demonstrated a non-significant reduction in anxiety and depression following PFCC.^37^

Regarding the duration of ICU stay, our study did not find a significant association with PFCC interventions, which aligns with the results of other studies.^38^, ^39^, ^40^, ^41^ Some studies have also indicated that PFCC can alleviate depression and anxiety even among family members participating in the intervention.

Our study has several strengths. It is one of the few studies that has attempted to generate evidence on the effectiveness of PFCC interventions on various ICU outcomes. Compared to previous reviews on the same topic, our review is more comprehensive as we included newer research articles with diverse outcomes and a larger sample size. All studies were independently screened and assessed for risk of bias by two authors. We exclusively included RCTs, further enhancing the quality of the pooled evidence.

However, our review also possesses several limitations.

1. Risk of bias in included studies: The review acknowledges that all included studies were assessed to have either a high or unclear risk of bias. Five out of the eleven studies had a high risk of bias concerning blinding, while the remaining six had an unclear risk due to insufficient information on blinding and outcome assessment. This high risk of bias significantly limits the validity and reliability of the meta-analysis findings.
2. Limited scope of databases: The systematic search was conducted only in three databases: Medline, Google Scholar, and ScienceDirect. Key databases such as Cochrane Central Register of Controlled Trials (CENTRAL) and EMBASE were not included. This restricted search may have resulted in the omission of relevant studies, potentially introducing selection bias.
3. Language bias: The review only included studies reported in English. This introduces language bias and may have excluded high-quality studies published in other languages, potentially impacting the comprehensiveness of the review.
4. Inconsistent outcome measures: Different studies utilized various tools and scales to measure the same outcomes (e.g., anxiety, depression). Although the authors pooled estimates using a weighted mean difference, the inconsistency in outcome measures could contribute to the observed heterogeneity and affect the validity of the pooled results.
5. Heterogeneity among studies: There was significant heterogeneity observed in the meta-analysis, particularly concerning the effects on delirium (I^2^ = 86.8%) and length of ICU stay (I^2^ = 97.8%). Such substantial heterogeneity suggests variability in study designs, populations, interventions, and outcome assessments, which complicates the interpretation of pooled effects and reduces the overall strength of the conclusions.
6. Focus on freely available full-text articles: The inclusion criteria were limited to freely available full-text articles. This restriction may have excluded potentially relevant studies not freely accessible, contributing to selection bias and reducing the comprehensiveness of the review.
7. Limited exploration of patient characteristics: The review did not account for the primary diagnoses or characteristics of the ICU patients in the included studies. Differences in patient demographics and clinical conditions could influence outcomes, and without this consideration, the applicability of the review's findings to diverse patient populations is limited.
8. Lack of sensitivity analysis: No sensitivity analysis was performed to assess the robustness of the findings. Given the high risk of bias in many studies, a sensitivity analysis could have provided valuable insights into how different studies or methodological choices affect the overall results.

While the systematic review provides valuable insights into the potential benefits of Patient and Family-Centered Care interventions in reducing delirium among ICU patients, the findings are substantially limited by high risk of bias, language restrictions, heterogeneity, and a limited scope of included studies. Future systematic reviews should aim to include a broader range of databases, consider studies in multiple languages, account for patient characteristics, and perform sensitivity analyses to enhance the reliability and applicability of the findings.

## Conclusion

This systematic review and meta-analysis suggests that PFCC interventions are effective in reducing the incidence of delirium among ICU patients. However, these interventions do not appear to significantly impact other psychological outcomes, such as anxiety and depression, or reduce the length of ICU stay. Given the high risk of bias and variability in study designs, future research should focus on high-quality, well-powered randomized controlled trials to more definitively determine the benefits of PFCC interventions in ICU settings. Further studies should also explore tailored interventions that address specific patient needs and consider the diverse settings of ICUs globally.

## Authors’ contributions

Conceptualization: Kaifang Cai. Data curation: Yangjin LV, Peng Li, Peng Li, Ronghui Li, Ting Zhang, Kaifang Cai. Formal analysis: Yangjin LV, Peng Li. Investigation: Ting Zhang. Methodology: Yangjin LV, Peng Li, Ronghui Li, Ting Zhang. Project Administration: Yangjin LV, Peng Li, Ronghui Li, Kaifang Cai. Resources: Peng Li. Software Yangjin LV, Ronghui Li. Supervision: Yangjin LV, Ting Zhang, Kaifang Cai. Validation: Yangjin LV, Peng Li, Ronghui Li, Ting Zhang, Kaifang Cai. Visualization: Peng Li, Kaifang Cai. Writing-original draft: Yangjin LV. Writing-review & editing: Yangjin LV, Peng Li, Ronghui Li, Ting Zhang, Kaifang Cai.

## Conflicts of interest

The authors declare no conflicts of interest.
